# Transmissible *Staphylococcus pseudintermedius* thwarts neutrophil extracellular trap-driven containment to promote invasive disease

**DOI:** 10.1080/22221751.2025.2482709

**Published:** 2025-04-02

**Authors:** Rita Haller, Yiyang Cai, Nicole de Buhr, Johanna C. Rieder, Dirk Schlüter, Claas Baier, Holger Rohde, Maren von Köckritz-Blickwede, Marius Vital, Volker Winstel

**Affiliations:** a Research Group Pathogenesis of Bacterial Infections; TWINCORE, Centre for Experimental and Clinical Infection Research, a joint venture between the Hannover Medical School and the Helmholtz Centre for Infection Research, Hannover, Germany; b Institute of Medical Microbiology and Hospital Epidemiology, Hannover Medical School, Hannover, Germany; c Department of Internal Medicine V, Universities of Giessen and Marburg Lung Center, University Hospital Giessen, Justus Liebig University Giessen, Member of the German Center for Lung Research (DZL), Giessen, Germany; d German Center for Infection Research (DZIF), Partner Site Giessen-Marburg-Langen, Justus Liebig University Giessen, Giessen, Germany; e Institute of Medical Microbiology, Justus Liebig University Giessen, Giessen, Germany; f Excellence Cluster Cardio-Pulmonary Institute (CPI), Giessen, Germany; g Institute for Lung Health (ILH), Justus Liebig University Giessen, Giessen, Germany; h Institute of Biochemistry, University of Veterinary Medicine Hannover, Hannover, Germany; i Research Center for Emerging Infections and Zoonoses (RIZ), University of Veterinary Medicine Hannover, Hannover, Germany; j Department of Small Animal Medicine and Surgery, University of Veterinary Medicine Hannover, Hannover, Germany; k Cluster of Excellence-Resolving Infection Susceptibility (RESIST), (EXC 2155), Hannover Medical School, Hannover, Germany; l Institute of Medical Microbiology, Virology and Hygiene, University Medical Center Hamburg-Eppendorf, Hamburg, Germany

**Keywords:** *Staphylococcus pseudintermedius*, zoonosis, NETosis, immune evasion, nuclease

## Abstract

Methicillin-resistant *Staphylococcus pseudintermedius* (MRSP) is an emerging zoonotic pathogen that causes a variety of clinical diseases in mammalian hosts. While it frequently causes infections in dogs and other domestic animals, accumulating evidence indicates that zoonotic spillover and cross-species transmission events favour local and invasive *S. pseudintermedius* infections in humans. However, immuno-evasive maneuvers that shape *S. pseudintermedius* pathogenicity and survival in diseased hosts remain enigmatic. Powered by multi-tech imaging and a mouse model of bloodstream infection, we illustrate that *S. pseudintermedius* adopted a virulence mechanism from predominant bacterial pathogens to surmount neutrophilic responses and neutrophil extracellular trap (NET)-mediated killing. Specifically, release of NucB, a thermostable nuclease, helps MRSP coping with the antimicrobial and pathogen-immobilizing properties of NETs and even promotes intra-neutrophil survival upon phagocytosis, thereby contributing to *S. pseudintermedius* pathogenesis and persistence within hepatic abscesses. Combined with the analysis of genetically distinct human clinical isolates, all of which display nuclease activity and features of resistance to NETosis-induced killing, our data highlight how zoonotic staphylococci overcome innate immune responses and concurrently uncover a mechanism that may exacerbate animal-borne MRSP infections in humans.

Since its initial description in 2005, *Staphylococcus pseudintermedius* has emerged as an underestimated zoonotic infectious agent [1–3]. Although representing an important canine pathogen, this microbe more and more frequently causes infections in humans that range from skin and soft tissue infections (SSTI) to fatal invasive diseases including endocarditis, pneumonia, or bacteremia [1,3,4]. Of note, mounting evidence indicates that not only the global spread of methicillin-resistant *S. pseudintermedius* (MRSP) but also steadily increasing epidemiological spillover events between colonized or diseased vertebrate animals and humans favour *S. pseudintermedius* infections in immunocompromised patients or otherwise healthy individuals [1,3,4], underpinning the pathogenic and pandemic potential of this microbe.

Regardless of the host or type of disease, *S. pseudintermedius* infections are characterized by strong inflammatory responses and the recruitment of neutrophils to infectious foci [5,6]. This becomes particularly evident in case of SSTIs or abscess formation in organ tissues upon bloodstream infection [5–7]. Nonetheless, neutrophil-driven pathogen control appears insufficient to clear *S. pseudintermedius* infections implying for the presence of effective mechanisms that foster immune escape and dissemination of disease. Specifically, *S. pseudintermedius* may have adopted conserved immuno-evasive tactics from high-priority pathogens to evade from neutrophilic responses and particularly neutrophil extracellular traps (NETs), a pathogen-capturing and antimicrobial matrix that is expelled from neutrophils in response to microbial invaders [8,9]. If so, *S. pseudintermedius* may not only better survive during initial infection stages but also escape into healthy tissues or circulating body fluids, thereby promoting the establishment of invasive disease. To examine this possibility, human neutrophils were initially exposed to a patient-derived MRSP isolate (DSM 25713; Table S1) or filter-sterilized culture supernatants derived thereof and analyzed for NETs release via SYTOX Green staining or immunofluorescence microscopy, applying an antibody targeting the NET-specific marker protein myeloperoxidase (MPO). As expected, neutrophils sensed MRSP and formed NETs, albeit these structures disappeared over time, suggesting that elements of the *S. pseudintermedius* secretome may contain factors that break down NETs following their release from neutrophils (Figure S1). To analyze this in more detail, we asked if *S. pseudintermedius* may utilize extracellular nucleases to degrade NETs in a manner similar to other clinically relevant pathogens such as *Staphylococcus aureus* [10]. Human neutrophils were therefore stimulated with the NETosis-triggering agent phorbol-12-myristate-13-acetate (PMA) prior exposure to *S. pseudintermedius* wild type or a variant lacking the nuclease-encoding gene *nucB* [5]. While wild-type *S. pseudintermedius* and the complemented *nucB* deletion mutant disrupted NETs in this approach, no degradation was visible in samples that were treated with the *nucB*-deficient variant (Figure 1(A–I)). Likewise, only *nucB*-containing culture filtrates, but not a *S. pseudintermedius* mutant lacking another putative nuclease (*nucA*) [5], degraded NETs (Figures S2 and S3). Moreover, real-time visualization of NETosis uncovered that wild-type MRSP-derived supernatants diminished PMA-induced NET formation in a time-dependent manner when compared to samples treated with culture filtrates obtained from the *nucB* deletion mutant or PMA only (Figure 1(J)). This is further supported by the notion that only the addition of NucB-containing culture filtrates to mature NETs several hours post PMA stimulation forced their disruption (Figure 1(J)). Similar findings were also obtained when netting neutrophils or mature NETs were exposed to recombinant NucB (rSpNucB) (Figure 1(K–P)), which, together with the observation that even canine neutrophil-derived NETs were broken down by NucB (Figure S4), suggests that *S. pseudintermedius* not only exploits this enzyme to degrade NETs but also to overcome their pathogen-immobilizing and antimicrobial features. To test this conjecture, green fluorescent protein-expressing MRSP were incubated with NETs-expelling neutrophils and examined for entrapment via immunofluorescence microscopy. Compared to NETs-evading wild-type *S. pseudintermedius*, *nucB*-deficient staphylococci failed to disrupt NETs and thus were captured within the DNA matrix (Figure S5). PMA-activated human neutrophils were also challenged with the *S. pseudintermedius* strain panel in the presence of the phagocytosis-inhibiting agent cytochalasin D, which revealed that only wild-type MRSP, but not its *nucB*-deficient variant, were refractory to NET-mediated killing (Figure 1(Q)). Since not all neutrophils eject NETs in response to appropriate stimuli, and a balanced ratio of netting and phagocytosing neutrophils is required for efficient elimination of microorganisms [8], non-PMA stimulated neutrophils were also exposed to the *S. pseudintermedius* panel in a cytochalasin D-free environment. Once again, lack of *nucB* rendered MRSP more susceptible to NETs- and neutrophil-mediated killing, highlighting that NucB not only helps *S. pseudintermedius* coping with the adhesive and antimicrobial properties of NETs but may also contribute to intra-neutrophil survival of MRSP following phagocytosis (Figure 1(Q)).

**Figure 1.**
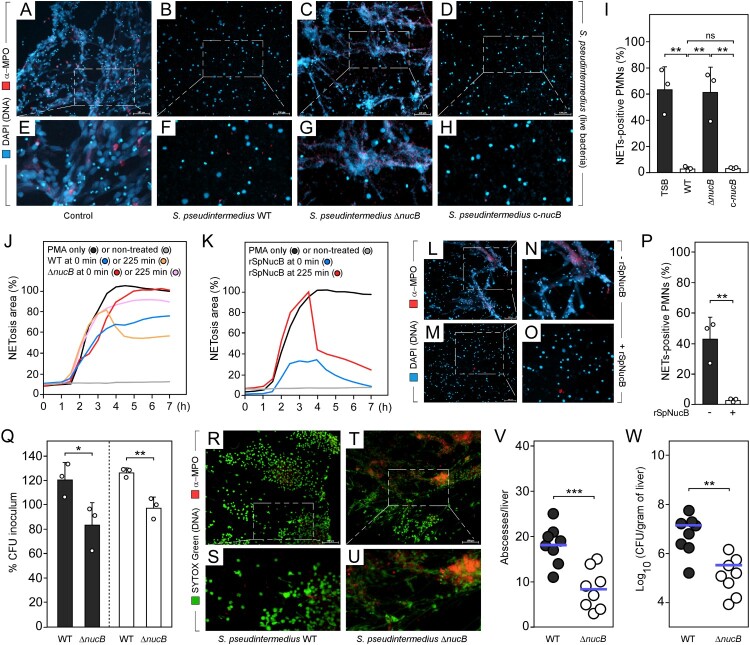
*S. pseudintermedius* exploits NucB-driven breakdown of NETs for intra-host survival. (A–H) Immunofluorescence imaging of human NETs treated with wild-type (WT) *S. pseudintermedius* DSM 25713, its *nucB* (Δ*nucB*) or complemented *nucB* mutant (c-*nucB*), or sterile TSB medium (control). NETosis was initiated by PMA treatment for 180 min prior exposure to staphylococci. (I) Quantification of NETosis rates of MRSP-treated NETs. (J, K) Real-time analysis of NETosis. Samples received PMA at *t* = 0 min and were either exposed to staphylococcal culture filtrates (J) or rSpNucB (K). Treatment was started immediately or initiated 225 min post stimulation. Shown are medians of technical duplicates out of three independent donors. (L-P) Immunofluorescence imaging (L-O) and NETosis quantification rates (P) of rSpNucB-treated NETs. (Q) Staphylococcal survival rates within human NETs or neutrophils. PMA-triggered neutrophils were exposed to cytochalasin D and challenged with the *S. pseudintermedius* strain panel for 60 min (black columns). Neutrophil-mediated killing of staphylococci (white columns) was conducted without adding PMA and cytochalasin D for 60 min. Data were recorded as percent inoculum. (R-U) Immunofluorescence imaging of hepatic abscess aspirates extracted 5 days after intravenous injection of WT *S. pseudintermedius* DSM 25713 or its Δ*nucB* mutant into C57BL/6 mice. (V, W) Enumeration of surface abscesses (V) and staphylococcal loads (W) in livers after intravenous injection of WT *S. pseudintermedius* DSM 25713 (filled circles) or its Δ*nucB* mutant (open circles) into female C57BL/6 mice (*n* = 8). Bacterial burden was enumerated as log_10_ CFU per gram of tissue at 5 days post-infection. Horizontal blue bars represent the mean values of visible abscesses per liver (V) or CFU counts in each cohort (W). NETs were visualized by using an antibody against myeloperoxidase (α-MPO; red) along with DAPI (blue; A–H and L–O) or SYTOX Green (green; R–U) to stain DNA. Magnifications of boxed areas are indicated (E–H, N–O, S, U). Representative images are shown (scale bars, 100 µm). Primary cell experiments included three independent donors. Data are the mean (± standard deviation [SD]) values from three biologically independent determinations (I, P–Q). Statistically significant differences were analyzed by one-way (I) analysis of variance (ANOVA) followed by Tukey’s multiple-comparison test, via a two-tailed Student’s *t*-test (P–Q, V), or by using the Mann-Whitney test if data did not pass normality distribution tests (W); ns, not significant (*P* ≥ 0.05); *, *p* < 0.05; **, *p *< 0.01; ***, *p* < 0.001.

Having demonstrated that MRSP overcomes NETs-mediated killing via NucB secretion, we next sought to assess how this mechanism impacts *S. pseudintermedius* pathogenesis *in vivo*. To investigate this, we analyzed NETosis in a murine model of bloodstream infection and dissected livers from wild-type MRSP- or *nucB*-infected animals for the collection of abscess aspirates, which were subjected to an immunofluorescence microscopy-based approach. Notably, MPO-positive NETs were hardly detectable in pus obtained from wild-type MRSP*-*challenged mice (Figure 1(R–S)). Conversely, purulent materials derived from *nucB* deletion mutant-infected animals contained MPO-positive DNA fibres, which demonstrates NucB-facilitated degradation of NETs that form in response to MRSP infection (Figure 1(T–U)). In this regard and in line with our earlier findings [5], we further note that abscessation and bacterial loads were strongly diminished in livers of animals that have been challenged with the *nucB* deletion mutant (Figure 1(V–W)). This is likely linked to enhanced killing of the *nucB*-deficient variant within NETs as growth of the mutant was unaffected under laboratory conditions when compared to its parental strain (Figure S6). Thus, NucB-induced collapse of NETs promotes intra-host survival of MRSP and may therefore represent a universal feature that helps most, if not all, clinical *S. pseudintermedius* isolates to bypass NETs-mediated killing. To examine this, we finally exposed NETs-ejecting neutrophils to a panel of patient-derived and *nucB*-proficient clinical *S. pseudintermedius* isolates, all of which displayed a high degree of phylogenetical diversity when compared to a global collection of *S. pseudintemedius* isolates of human and zoonotic origin (Table S2; Figure S7), for subsequent analysis of bacterial survival rates. Similar to the assay-internal reference MRSP strain DSM 25713, which required NucB to escape from NETs (Figure 1(Q)), all human *S. pseudintermedius* isolates as well as the canine isolate ED99 were refractory to the antimicrobial properties of NETs (Table S2). Although we cannot exclude the possibility that auxiliary mechanisms may contribute to survival of zoonotic staphylococci within NETs, these data collectively imply that *S. pseudintermedius* utilizes NucB to antagonize neutrophil responses and NETs-induced killing.

## Discussion

Despite being a zoonotic pathogen of canine origin, current understanding of *S. pseudintermedius* virulence determinants that promote pathogenesis and animal-borne diseases in humans is extremely limited. This is probably a result of inefficient toolkits for genetic engineering of clinical isolates along with the lack of proper *in vivo* models and information on phagocyte responses that occur during *S. pseudintermedius* infections. Using a multipronged approach, we report that neutrophils expel antimicrobial NETs upon sensing of MRSP and its exoproducts. The exact mechanism capable of priming MRSP-driven NETosis, however, remains unclear but likely depends on toll like receptor 2 (TLR2) and its capacity to sense staphylococcal lipoproteins [11,12]. Since TLR2-mediated signaling is further linked to vital NETosis, a process where neutrophils maintain their phagocytic activity [8], NETs-forming neutrophils may attempt to immobilize and clear *S. pseudintermedius* at primary infection sites without undergoing suicide. Nonetheless, *S. pseudintermedius* escapes from NET-facilitated containment and killing via NucB secretion, which promotes virulence and the establishment of suppurative tissue abscesses. Accordingly, release of NucB is also expected to exacerbate neutrophil-dominated SSTI in humans following zoonotic transmission or, *vice versa*, may impact infection outcomes in animal hosts. Moreover, NucB may also support MRSP to initiate additional immuno-evasive maneuvers that foster persistent infection and pathogen dispersal. In fact, previous studies indicate that disruption of NETs lowers immune cell networking and the bactericidal activity of macrophages toward *S. aureus* [13]. Concurrently, nuclease-mediated digestion of NETs, together with the activity of peptidoglycan-anchored nucleotidases, results in the biogenesis of effector-nucleosides that prevent phagocyte entry into abscesses [14,15]. Such strategy may even raise the possibility that MRSP selectively rather than accidentally trigger neutrophils to expel NETs. Consequently, development of small molecule inhibitors or monoclonal antibodies targeting NucB could help to ameliorate infectious diseases caused by drug-resistant *S. pseudintermedius* in humans, canines, or other domesticated animals.

## Supplementary Material

Supplemental material Haller et al.pdf

## Data Availability

All relevant data are within the paper and its Supporting Information files.
