# Supplementary material for: Transmissible Staphylococcus pseudintermedius thwarts neutrophil extracellular trap-driven containment to promote invasive disease
Source: Emerg Microbes Infect. 2025 Apr 2;14(1):2482709. doi: 10.1080/22221751.2025.2482709 (PMC12001851; doi:10.1080/22221751.2025.2482709)
Supplement: Supplemental material Haller et al.pdf [file TEMI_A_2482709_SM1106.pdf]

Supplemental Materials - *Haller et al.*

Transmissible *Staphylococcus pseudintermedius* thwarts neutrophil extracellular trap-driven containment to promote invasive disease

Authors: Rita Haller[a,b], Yiyang Cai[a,b,c,d,e,f,g], Nicole DeBuhr[h,i], Johanna C. Rieder[j], Dirk Schlüter[b,k], Claas Baier[b], Holger Rohde[l], Maren von Köckritz-Blickwede[h,i], Marius Vital[b], Volker Winstel[a,b,c,d,e,f,g*]

[a] Research Group Pathogenesis of Bacterial Infections; TWINCORE, Centre for Experimental and Clinical Infection Research, a joint venture between the Hannover Medical School and the Helmholtz Centre for Infection Research, Hannover, Germany

[b] Institute of Medical Microbiology and Hospital Epidemiology, Hannover Medical School, Hannover, Germany

[c] Department of Internal Medicine V, Universities of Giessen and Marburg Lung Center, University Hospital Giessen, Justus Liebig University, Member of the German Center for Lung Research (DZL), Giessen, Germany

[d] German Center for Infection Research (DZIF), Partner Site Giessen-Marburg-Langen, Justus Liebig University Giessen, Giessen, Germany

[e] Institute of Medical Microbiology, Justus Liebig University Giessen, Giessen, Germany

[f] Excellence Cluster Cardio-Pulmonary Institute (CPI), Giessen, Germany

[g] Institute for Lung Health (ILH), Justus Liebig University, Giessen, Germany

[h] Institute of Biochemistry, University of Veterinary Medicine Hannover, Hannover, Germany

[i] Research Center for Emerging Infections and Zoonoses (RIZ), University of Veterinary Medicine Hannover, Hannover, Germany

[j] Department of Small Animal Medicine and Surgery, University of Veterinary Medicine Hannover, Hannover, Germany

[k] Cluster of Excellence-Resolving Infection Susceptibility (RESIST), (EXC 2155), Hannover Medical School, Hannover, Germany

[l] Institute of Medical Microbiology, Virology and Hygiene, University Medical Center Hamburg-Eppendorf, Hamburg, Germany

* Address correspondence to Volker Winstel: volker.winstel@innere.med.uni-giessen.de

Supplemental Materials and Methods

Bacterial strains. All bacterial strains used in this study are listed in Table S1 and were grown in tryptic soy broth (TSB) or lysogeny broth (LB) at permissive temperatures. Growth media were supplemented with appropriate antibiotics (ampicillin 100 µg/ml; chloramphenicol 10 µg/ml).

Molecular genetics. The generation of the *S. pseudintermedius* mutant panel is described elsewhere [1]. To visualize staphylococci within NETs, *S. pseudintermedius* wild type or the *nucB* deletion mutant were equipped with the pC183-S3 plasmid harboring the green fluorescent protein (GFP) protein-encoding gene *gfp* [2]. pC183-S3 was isolated from *S. aureus* strain SA113, transferred to the *S. pseudintermedius* strain panel via electroporation as described elsewhere [3], and maintained in the presence of chloramphenicol (10 µg/ml).

Isolation of primary cells. Human primary neutrophils were isolated by a standard density gradient separation method according to standard laboratory protocols. Canine primary neutrophils were isolated by density gradient centrifugation in combination with a hypotonic lysis of the erythrocytes according to the methodology described by Steffensen et al. [4].

Protein purification. Recombinant NucB was purified as described elsewhere [1]. In brief, a recombinant and glutathione S-transferase (GST)-tagged variant of *S. pseudintermedius* NucB (rSpNucB) was expressed in *E. coli* BL21 using the pGEX-2T system (GE Healthcare). Next, glutathione S-transferase affinity chromatography was used to purify the protein according to a published approach [1]. The N-terminal GST tag was removed via thrombin cleavage. Benzamidine sepharose beads (GE Healthcare) were used to remove thrombin from the sample. Purified rSpNucB was analyzed by Coomassie-stained SDS-PAGE according to standard laboratory protocols.

Induction and visualization of NETs. To induce NET formation, 2.5 x 10^5 freshly isolated primary neutrophils were resuspended in RPMI 1640 medium containing 2% human serum albumin (HSA) and 100 nM of the NETosis-stimulating agent phorbol-12-myristate-13-acetate (PMA). Cells were then seeded in 24-well plates or onto glass microscopy coverslips and incubated for 3 h at 37°C under 5% CO_2. Alternatively, mid-log phase-derived and filter-sterilized *S. pseudintermedius* culture supernatants (final conc. 10%) were added directly to neutrophils and incubated at 37°C under 5% CO_2 as indicated in the figures. For experiments involving live bacteria, NET formation was stimulated for 60 or 120 min at 37°C under 5% CO_2 by adding 3.7 x 10^7 colony-forming units (CFU) of mid-log phase grown *S. pseudintermedius* to the neutrophil suspension. Following incubation, the supernatants or bacteria were removed, and the samples were washed once with PBS. Next, neutrophils and NETs were stained with SYTOX Green (167 nM) for 15 min in the dark, washed

with PBS, and analyzed via fluorescence microscopy. Alternatively, the samples were fixed for 15 min at RT by using 4% paraformaldehyde solution, washed once in PBS, and subjected to the immunofluorescence staining procedure.

NET entrapment assay. NET entrapment assays were performed as described before, with minor modifications [5]. In brief, 2.5 x 10^5 freshly isolated primary neutrophils were resuspended in RPMI 1640 medium containing 2% HSA and 100 nM of the NETosis-stimulating agent PMA. Cells were then seeded onto glass microscopy coverslips and incubated for 3 h at 37°C under 5% CO_2. Next, 3.7 x 10^7 CFU of mid-log phase-grown *S. pseudintermedius* carrying the pC183-S3 plasmid were directly added to the neutrophil suspension, briefly centrifuged (5 min at 200 x g, RT), and further incubated for 90 min at 37°C under 5% CO_2. Coverslips were washed to remove non-entrapped bacteria, fixed for 15 min at RT by using 4% paraformaldehyde solution, washed once more, and subjected to the immunofluorescence staining procedure.

NET degradation assay. 2.5 x 10^5 freshly isolated primary neutrophils were stimulated with PMA as described above, exposed to 3.7 x 10^7 CFU of mid-log phase-derived *S. pseudintermedius* or filter-sterilized culture supernatants derived thereof (final conc. 10%), and incubated at 37°C under 5% CO_2 for 90 min. Following this step, culture supernatants were removed. Subsequently, samples were fixed for 15 min at RT by using 4% paraformaldehyde solution and subjected to the immunofluorescence staining procedure. Alternatively, PMA-triggered neutrophils were incubated for 30 min at 37°C under 5% CO_2 in RPMI 1640 supplemented with 2% HSA, 60 mM Tris-HCl (pH 7.4), 0.6 mM $MgCl_2$, 0.6 mM $CaCl_2$, and rSpNucB (10 ng/µl). Samples were then washed once in PBS and subjected to the immunofluorescence staining procedure.

NET and neutrophil killing assay. 2.5 x 10^5 freshly isolated primary neutrophils per well were mixed with RPMI 1640 medium containing 2% HSA and seeded into a wells of a tissue culture-treated 24-well plate. Following a brief incubation step at 37°C (60 min, 5% CO_2), neutrophils were exposed to a mixture of 100 nM PMA and the phagocytosis-inhibiting substance cytochalasin D (10 µg/ml). Samples were further incubated for 30 min at 37°C under 5% CO_2 and then infected with 5.0 x 10^5 CFU of mid-log phase-grown *S. pseudintermedius* wild type or the *nucB* deletion mutant. Alternatively, neutrophils were directly infected with staphylococci in the absence of PMA and cytochalasin D. Next, the 24-well plates were briefly centrifuged at RT (250 x g, 5 min) and incubated for 60 min at 37°C under 5% CO_2. Finally, samples were mixed in a 1:1 ratio with sterile lysis buffer (PBS containing 1.0% saponin) and incubated for 10 min at 37°C to lyse all remaining eukaryotic cells. Subsequently, serial dilutions were prepared and plated onto TSA plates to determine bacterial survival rates.

Real-time imaging of NETosis. Real-time imaging of NET formation was performed by using an IncuCyte SX5 system (Satorius) and a previously described approach with minor modifications [6,7]. In brief, 4.0 x 10^4 freshly isolated primary human neutrophils per well (96-well plate) were mixed with 100 µl of phenol red-free RPMI 1640 medium containing 2% HSA, SYTOX Green (1 µM), and 100 nM PMA. Subsequently, the 96-well plate was transferred into the IncuCyte reader and incubated at 37°C under 5% CO_2. Real-time imaging was performed by using appropriate microscopy settings (objective 20X, acquisition time 300 ms). Next, raw images were processed using the following filters settings: *Phase channel*: segmentation adjustment 0.2; hole Fill 1; adjust size +2 pixels; minimal area 100 $µm^2$; maximal eccentricity 0.99; *Green channel*: Top-hat segmentation mode; Radius: 10 µm; Threshold (Green Calibrated Unit, GCU) 1.0; Edge sensitivity, -10; Hole fill: 100 $µm^2$; Minimal area 100 $µm^2$. Images were taken at 30 min intervals as indicated in appropriate figures. Where indicated, PMA-treated cells were either immediately exposed to filter-sterilized staphylococcal culture supernatants (5 %) or treated with culture filtrates 225 min post PMA stimulation. Alternatively, samples received rSpNucB (20 ng/µl) at the beginning of the experiment or 225 min post PMA stimulation. Raw data were processed by using the IncuCyte GUI 2023A pipeline. To calculate neutrophils undergoing NETosis, data obtained from the green channel (total fluorescence) was divided by the data derived from the phase channel (total cell area) at time point 0 min and expressed as a percentage.

Immunofluorescence staining. To visualize NETs, coverslips were initially washed once with PBS solution and then blocked at RT for 60 min by adding blocking buffer (PBS supplemented with 10% FCS and 0.1% Triton X-100). Immunofluorescence staining was carried out by using an antibody against MPO (α-MPO, ab208670, human samples; α-MPO, RB-373-A, canine samples), followed by a fluorescently labelled secondary antibody. Following this procedure, coverslips were washed three times in PBS and double-distilled water, mounted with Prolong Gold reagent containing 4',6'-diamidino-2-phenylindole (DAPI), and examined by using a Zeiss Apotome 2 microscope (Zeiss). Four random fields per condition were used for the quantification of NETosis, which was calculated by counting netting neutrophils (neutrophils displaying extracellular DNA fibers together with decondensed nuclei and co-localized MPO) along with the total number of cells. NETosis rates are given as a percentage relative to the total number of neutrophils.

Animal work. C57BL/6 mice were purchased from Janvier Laboratories and kept under specific pathogen-free conditions in our central mouse facility (TWINCORE, Center for Experimental and Clinical Infection Research, Hannover, Germany). For *in vivo* experiments, wild-type *S. pseudintermedius* DSM 25713 or its *nucB* deletion mutant were cultivated overnight in TSB, diluted 1:100 in TSB, and grown to an optical density (600 nm) of 0.5. Next, staphylococci were collected by a brief centrifugation step (10 min, RT, 8,000 × g), washed twice in sterile PBS, and adjusted to 10^8 CFU/ml. One hundred microliters of the resulting bacterial

suspension (10^7 CFU) were administered intravenously (lateral tail vein) into 6- to 8-weeks-old female mice. Five days post-infection, animals were killed. Livers were dissected and analyzed for surface abscesses. Subsequently, abscesses and derived purulent materials were removed, transferred onto microscope slides, and allowed to air dry. Samples were fixed at RT for 10 min by using ice-cold acetone, washed twice in PBS, and transferred into blocking solution (PBS containing 10% FCS and 0.1% Triton X-100) for 60 min. Next, washed slides were subjected to imaging procedure and stained by using a mixture of SYTOX Green (167 nM) and a primary antibody against MPO (α-MPO, ab208670), followed by incubation with a fluorescently labelled secondary antibody. Slides were washed, mounted, and examined via microscopy as described above. Alternatively, dissected livers were homogenized in sterile PBS containing 0.1% Triton X-100. Serial dilutions were prepared and plated onto TSA plates to determine bacterial loads according to standard laboratory protocols.

Nuclease activity assays. To analyze nuclease activity of patient-derived *S. pseudintermedius* isolates, two microliters of staphylococcal overnight cultures were spotted onto DNase test agar plates (Oxoid) and incubated for 16 h at 37°C. DNase indicator plates were flooded with 1 N HCl and analyzed for clear zones around bacterial colonies that indicate nuclease-mediated hydrolysis of DNA.

Whole genome sequencing and bioinformatics analysis. DNA extraction, library generation, and sequencing on Illumina MiSeq (2x 250bp) was done as previously described [8]. Processing of raw reads was done according to Happ et al. [9] including quality filtering (fastp; v0.19.5, default mode), assembly (SPAdes; v3.15.5, careful mode), gene calling (prokka; v1.14.6, -fast mode) and calculation of the Average Nucleotide Identity (ANI) (fastANI; v1.33, default mode). A circularized dendrogram based on ANI results was constructed in R (4.2.2) with dendextend (v1.17.1) and circlize (v0.4.16); 49 genomes of human isolates downloaded from PATRIC, one strain derived from a dog (ED99), and the reference strain DSM 25713 were included for comparison reasons. Determination of the *nucB* gene was done via blasting (blastp; v2.6.0+) against reference sequences SPSE_1452 (KEGG). A similar approach was used for the detection of *mecA*. Raw sequences files are available at the European Nucleotide Archive (PRJEB83027).

Ethics statement. All animal experiments were conducted in accordance with the local animal welfare regulations reviewed by the institutional review board and the Niedersächsisches Landesamt für Verbraucherschutz und Lebensmittelsicherheit (LAVES) under the permission number 33.19–42502-04-20/3528. Human blood samples were obtained from adult, consenting healthy donors. Informed consent forms were obtained from all participants. These studies were reviewed and approved by the medical ethics committee of Hannover Medical School (Hannover, Germany) under the permission number

8831_BO_K_2019. Collection and scientific use of patient-derived bacterial isolates were approved by the medical ethics committee of Hannover Medical School (Hannover, Germany) under the permission number 11605_BO_K_2024. The collection of blood from healthy dogs was registered at the LAVES under the permission number 33.8-42502-05-21A609 and conducted in line with the recommendations of the German Society for Laboratory Animal Science (Gesellschaft für Versuchstierkunde) and the German Veterinary Association for the Protection of Animals (Tierärztliche Vereinigung für Tierschutz e. V.) (GV-SOLAS Gesellschaft für Versuchstierkunde. Available online: http://www.gv-solas.de) (accessed on 27 April 2021). All blood taking procedures were conducted with oral consent from the dog owners at the Department of Small Animal Medicine and Surgery and the Department of Biochemistry, University of Veterinary Medicine Hannover, Germany.

Statistical analysis. Statistical analysis was performed using GraphPad Prism (GraphPad Software, Inc., La Jolla, USA). Statistically significant differences were calculated by using statistical methods as indicated. *P* values < 0.05 were considered significant.

Table S1. Bacterial strains used in this study

Strain	Description	Reference
E. coli BL21 (DE3) pGEX-2T-*nucB*	BL21 bearing pGEX-2T-*nucB* expression plasmid	[1]
S. aureus SA113 pC183-S3	SA113 bearing the pC183-S3 GFP plasmid	[2]
S. pseudintermedius DSM 25713	Human clinical isolate, methicillin-resistant (obtained from DSMZ strain collection)	[10]
S. pseudintermedius Δ*nucA*	DSM 25713 Δ*nucA*	[1]
S. pseudintermedius Δ*nucB*	DSM 25713 Δ*nucB*	[1]
S. pseudintermedius Δ*nucB* pRB473-*nucB*	DSM 25713 Δ*nucB* complemented with pRB473-*nucB*	[1]
S. pseudintermedius DSM 25713 pC183-S3	DSM 25713 bearing the pC183-S3 GFP plasmid	This study
S. pseudintermedius Δ*nucB* pC183-S3	DSM 25713 Δ*nucB* bearing the pC183-S3 PFG plasmid	This study
S. pseudintermedius M002	Human clinical isolate, Hannover Medical School (Hannover, Germany)	This study
S. pseudintermedius M004	Human clinical isolate, Hannover Medical School (Hannover, Germany)	This study
S. pseudintermedius M649	Human clinical isolate, Hannover Medical School (Hannover, Germany)	This study
S. pseudintermedius H060	Human clinical isolate, University Medical Center Hamburg-Eppendorf (Hamburg, Germany)	This study
S. pseudintermedius ED99	Canine clinical isolate, UK	[11,12]

Table S2. Survival rates of clinical and NucB-proficient *S. pseudintermedius* in human NETs

S. pseudintermedius isolate	Infection origin	Geographic origin	*mecA*	*nucB*[a]	Nuclease activity[b]	Survival rate in human NETs (%)[c]
DSM 25713	Human	Italy	+	+	+	178.1 ± 55.19
M002	Human	Germany	-	+	+	184.5 ± 47.69
M004	Human	Germany	-	+	+	171.1 ± 36.71
M649	Human	Germany	-	+	+	164.9 ± 16.74
H060	Human	Germany	-	+	+	187.6 ± 46.23
ED99	Dog	UK	-	+	+	211.9 ± 54. 81

[a] All found with 100% coverage and >95% identity on the protein level; [b] Nuclease activity on DNase indicator agar plates; [c] Staphylococcal survival rates within NETs after 60 min of incubation. Data are the mean (± standard deviation [SD]) values from three biologically independent determinations and were recorded as percent inoculum. Primary cell experiments included three independent donors.

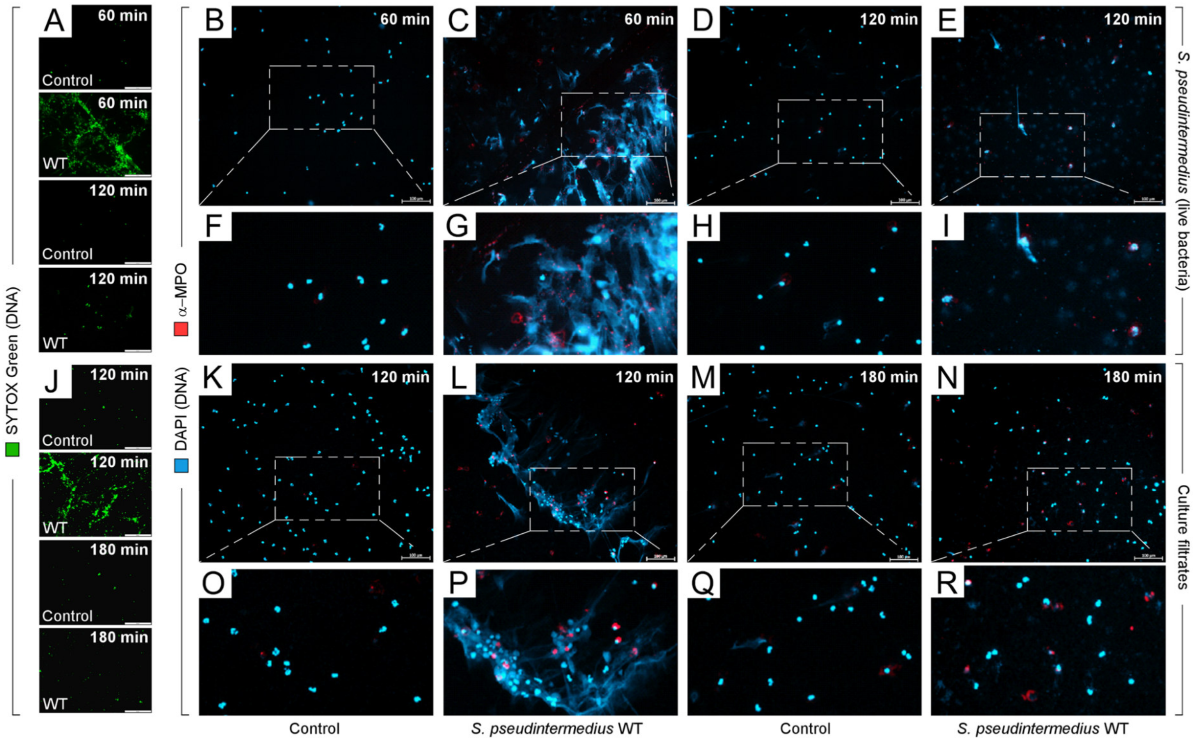

Figure S1. *S. pseudintermedius* shapes NET formation by a secretome-driven molecular switch. (A-R) Fluorescence microscopy images of human neutrophils forming NETs upon exposure to *S. pseudintermedius* DSM 25713 wild type (WT) (A-I) or culture filtrates derived thereof (J-R). Neutrophils were stimulated with viable staphylococci or exposed to filter-sterilized culture supernatants as indicated. Sterile TSB medium was used as a control. Magnifications of boxed areas are indicated (F-I and O-R). NETs were visualized by using SYTOX Green (A, J) or an antibody against myeloperoxidase (α-MPO; red) along with DAPI to stain DNA (blue) (B-I and K-R). White bars shown in the images depict 50 µm (A, J) or 100 µm (B-E and K-N) length. Representative images are shown.

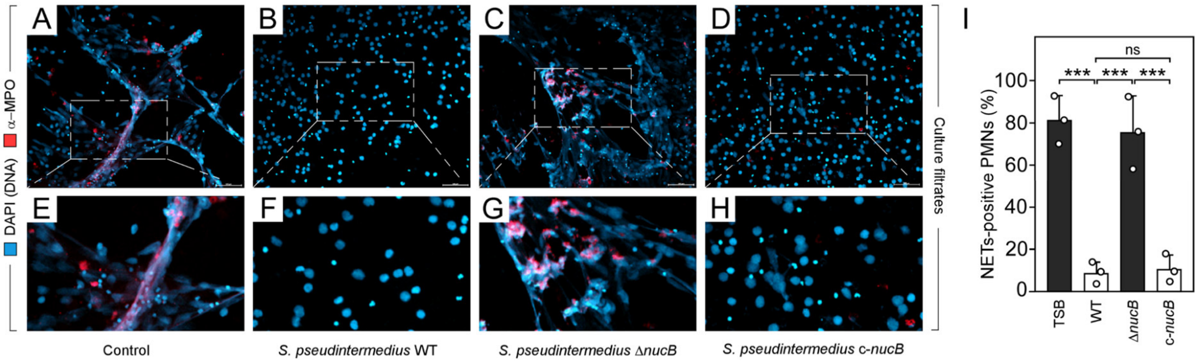

Figure S2. ***S. pseudintermedius*** **degrades NETs via NucB secretion. (A-H)** Immunofluorescence imaging of human NETs treated with culture filtrates derived from wild-type (WT) *S. pseudintermedius* DSM 25713, its *nucB* mutant (Δ*nucB*), or the complemented *nucB* variant (c-*nucB*). Formation of NETs was initiated by PMA treatment for 180 min prior exposure to culture supernatants. Sterile TSB medium was used as a control. Magnifications of boxed areas are indicated (E-H). **(I)** Quantification of NETosis rates of staphylococcal culture filtrate-treated NETs. NETs were visualized by using an antibody against myeloperoxidase (α-MPO; red) along with DAPI to stain DNA (blue). Representative images are shown (scale bars, 100 µm). Primary cell experiments included three independent donors. Data are the mean (± standard deviation [SD]) values from three biologically independent determinations. Statistically significant differences were analyzed by one-way (I) analysis of variance (ANOVA) followed by Tukey's multiple-comparison test; ns, not significant ($P \geq 0.05$); ***, p<0.001.

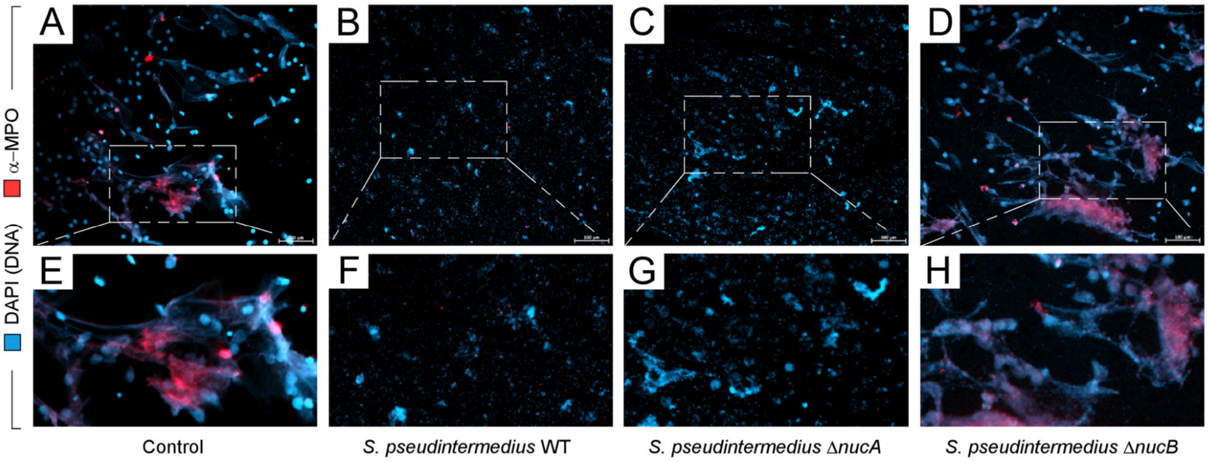

Figure S3. ***S. pseudintermedius*** **NucA does not contribute to degradation of NETs.** (A-H) Immunofluorescence imaging of human NETs treated with wild-type (WT) *S. pseudintermedius* DSM 25713, its *nucA* mutant (Δ*nucA*), or the *nucB* variant (Δ*nucB*). Formation of NETs was initiated by PMA treatment for 180 min prior exposure to staphylococci. Sterile TSB medium was used as a control. Magnifications of boxed areas are indicated (E-H). NETs were visualized by an antibody against myeloperoxidase (α-MPO; red) along with DAPI to stain DNA (blue). Representative images are shown (scale bars, 100 µm).

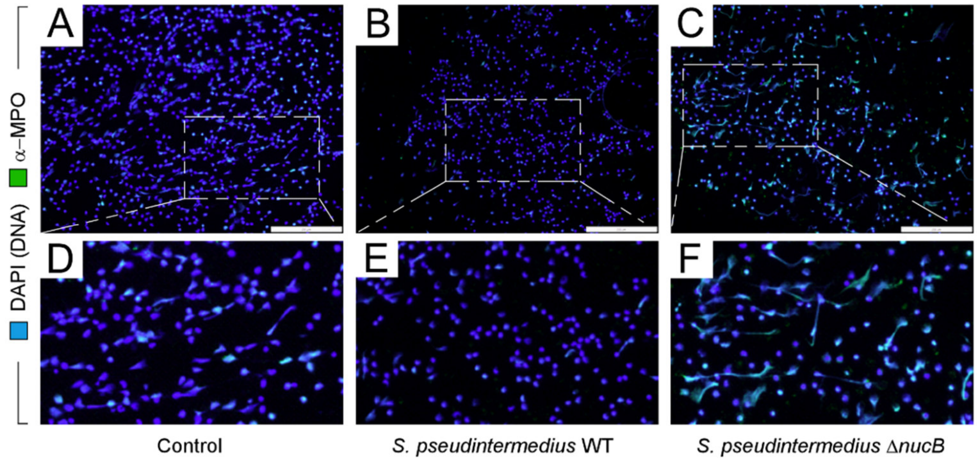

Figure S4. ***S. pseudintermedius*** **deploys NucB to degrade canine NETs. (A-F)** Immunofluorescence imaging of canine NETs treated with culture filtrates obtained from wild-type (WT) *S. pseudintermedius* DSM 25713 or its *nucB* mutant (Δ*nucB*). Formation of NETs was initiated by PMA treatment for 180 min prior exposure to bacterial culture supernatants. Sterile TSB medium was used as a control. Magnifications of boxed areas are indicated (D-F). NETs were visualized by using an antibody against myeloperoxidase (α-MPO; green) along with DAPI to stain DNA (blue). Representative images are shown (scale bars, 200 µm)

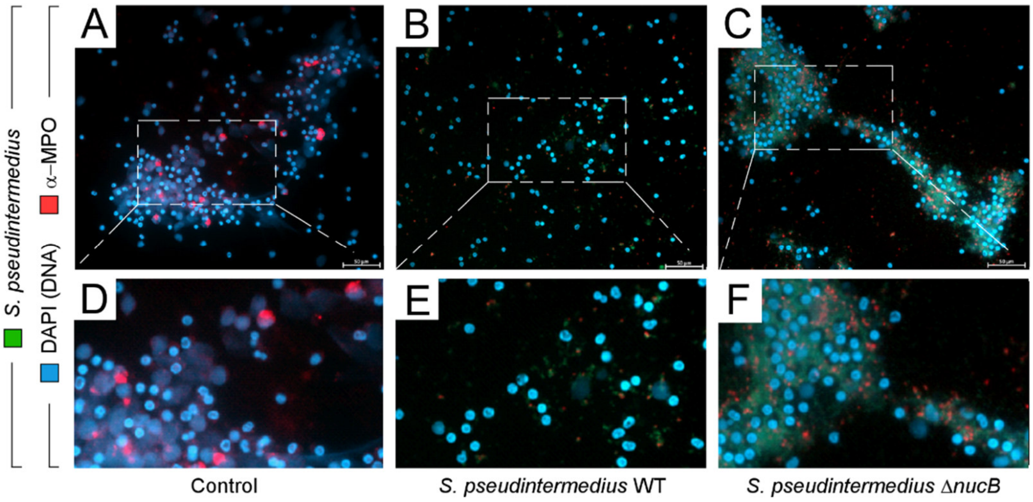

Figure S5. NucB shields *S. pseudintermedius* from NETs-mediated entrapment. (A-F) Immunofluorescence imaging of GFP-producing *S. pseudintermedius* (green) entrapped in human NETs. Formation of NETs was initiated by PMA treatment for 180 min prior exposure to wild-type (WT) *S. pseudintermedius* DSM 25713 or its *nucB* mutant (Δ*nucB*). Sterile TSB medium was used as a control. Magnifications of boxed areas are indicated (D-F). NETs were visualized by using an antibody against myeloperoxidase (α-MPO; red) along with DAPI to stain DNA (blue). Representative images are shown (scale bars, 50 µM).

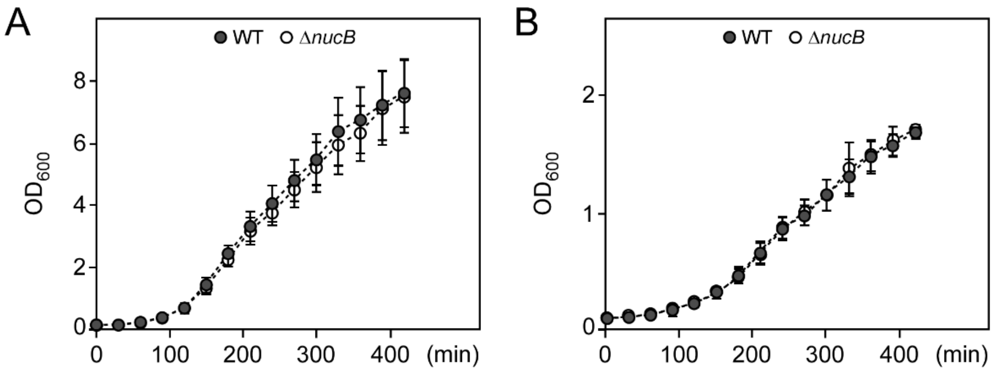

Figure S6. Lack of NucB is dispensable for *S. pseudintermedius* growth. (A, B) Analysis of growth characteristics of wild-type (WT) *S. pseudintermedius* DSM 25713 (filled circles) or its *nucB* mutant (Δ*nucB*) (open circles) in rich TSB (A) or chemically defined medium (RPMI 1640) (B). Optical density values at 600_{nm} are given. Data are the mean (± standard deviation [SD]) values from three biologically independent determinations. Statistical significance was determined by two-way analysis of variance (ANOVA) and Tukey's multiple comparison test; ns, not significant ($P \geq 0.05$). No significant differences were found.

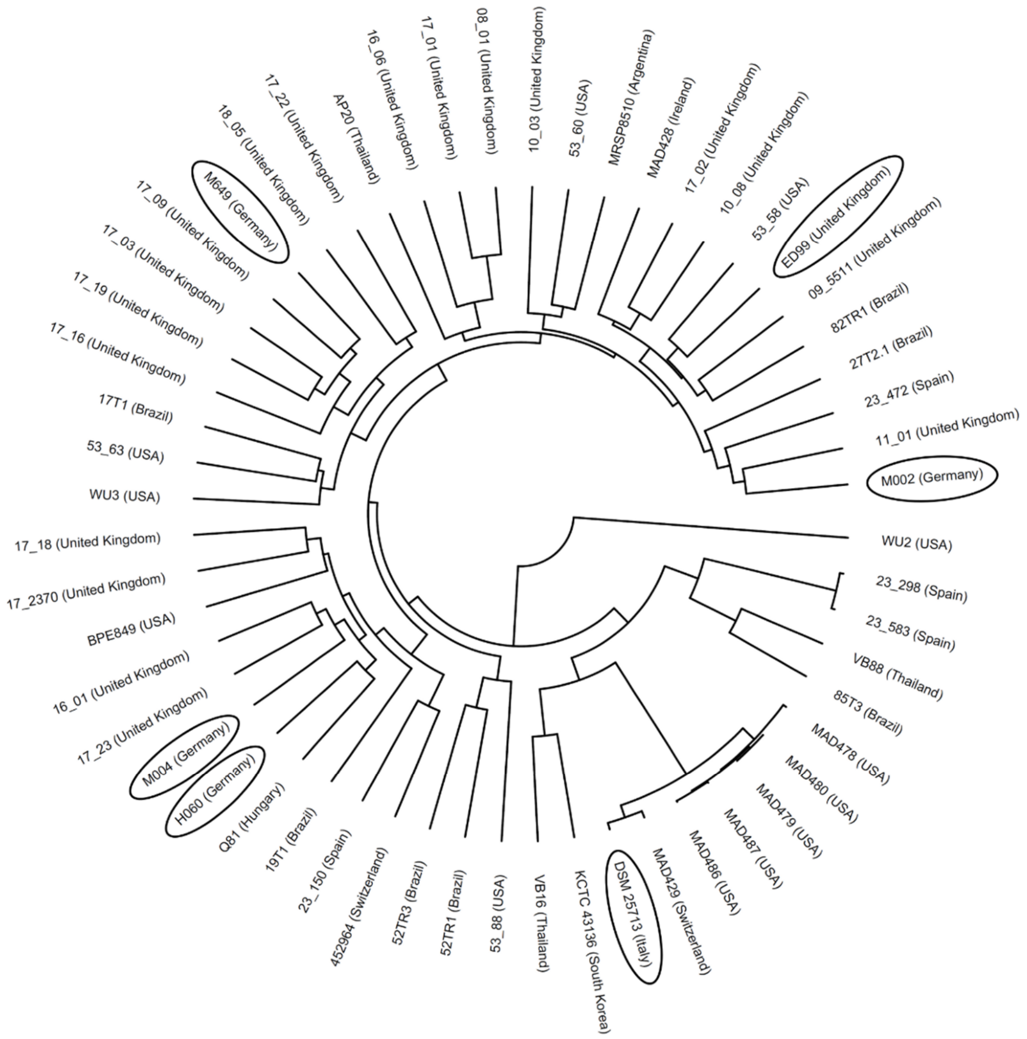

Figure S7. Dendrogram of *S. pseudintermedius* genomes based on whole genomes. The dendrogram is based on whole genome comparisons using Average Nucleotide Identity (ANI) calculations. The bacteria under study were compared to 49 human isolates from PATRIC, the reference strain DSM 25713, as well as one strain derived from a dog (ED99).

Supplemental References

[1] Bunsow D, Tantawy E, Ostermeier T, et al. Methicillin-resistant Staphylococcus pseudintermedius synthesizes deoxyadenosine to cause persistent infection. Virulence. 2021;12(1):989-1002.

[2] Burian M, Rautenberg M, Kohler T, et al. Temporal expression of adhesion factors and activity of global regulators during establishment of Staphylococcus aureus nasal colonization. J Infect Dis. 2010;201(9):1414-21.

[3] Schneewind O, Missiakas D. Genetic manipulation of Staphylococcus aureus. Curr Protoc Microbiol. 2014;32:Unit 9C 3.

[4] Steffensen N, Imker R, Lassnig S, et al. Methylprednisolone Induces Extracellular Trap Formation and Enhances Bactericidal Effect of Canine Neutrophils. Int J Mol Sci. 2021;22(14).

[5] Berends ET, Horswill AR, Haste NM, et al. Nuclease expression by Staphylococcus aureus facilitates escape from neutrophil extracellular traps. J Innate Immun. 2010;2(6):576-86.

[6] Gupta S, Chan DW, Zaal KJ, et al. A High-Throughput Real-Time Imaging Technique To Quantify NETosis and Distinguish Mechanisms of Cell Death in Human Neutrophils. J Immunol. 2018;200(2):869-879.

[7] Cockx M, Blanter M, Gouwy M, et al. The Antimicrobial Activity of Peripheral Blood Neutrophils Is Altered in Patients with Primary Ciliary Dyskinesia. Int J Mol Sci. 2021;22(12).

[8] Schafer F, Gorner P, Woltemate S, et al. The Resistance Mechanism Governs Physiological Adaptation of Escherichia coli to Growth With Sublethal Concentrations of Carbapenem. Front Microbiol. 2021;12:812544.

[9] Happ E, Schulze K, Afrin Z, et al. Gut microbiota-derived butyrate selectively interferes with growth of carbapenem-resistant Escherichia coli based on their resistance mechanism. Gut Microbes. 2024;16(1):2397058.

[10] Savini V, Barbarini D, Polakowska K, et al. Methicillin-resistant Staphylococcus pseudintermedius infection in a bone marrow transplant recipient. J Clin Microbiol. 2013;51(5):1636-8.

[11] Simou C, Thoday KL, Forsythe PJ, et al. Adherence of Staphylococcus intermedius to corneocytes of healthy and atopic dogs: effect of pyoderma, pruritus score, treatment and gender. Vet Dermatol. 2005;16(6):385-91.

[12] Bannoehr J, Ben Zakour NL, Waller AS, et al. Population genetic structure of the Staphylococcus intermedius group: insights into agr diversification and the emergence of methicillin-resistant strains. J Bacteriol. 2007;189(23):8685-92.
